# The Left Atrial Septal Pouch: A New Stroke Risk Factor?

**DOI:** 10.1007/s12975-020-00864-3

**Published:** 2021-01-04

**Authors:** Ruchi Kapoor, Lara Wadi, Brian Becerra, Michael Eskander, Ali Razmara, Dawn Lombardo, Annlia Paganini-Hill, Jin Kyung Kim, Mark Fisher

**Affiliations:** 1 Department of Medicine, Cardiology, University of Washington, Seattle, WA USA; 2 Department of Medicine, Cardiology, University of California Irvine, Orange, CA USA; 3 Department of Neurology, University of California Irvine, Orange and Irvine, CA USA; 4 Department of Medicine, Duke University, Durham, NC USA; 5 Department of Medicine, Cardiology, University of California San Diego, San Diego, CA USA; 6 Department of Neurology, UC Irvine Medical Center, 101 The City Drive South, Building 55, Room 121, Orange, CA 92868 USA

**Keywords:** Cryptogenic stroke, Inter-atrial septum, Ischemic stroke, Left atrial septal pouch, Transesophageal echocardiogram

## Abstract

The left atrial septal pouch (LASP) occurs due to incomplete fusion of septa primum and secundum at the inter-atrial septum, creating an open flap that may serve as a thromboembolic source. Prior studies have demonstrated increased prevalence of LASP in cryptogenic strokes. The aim of the current study was to validate the above findings in a separate, larger group of stroke and non-stroke patients. We examined transesophageal echocardiograms (TEEs) performed between July 2011 and December 2018. LASP prevalence was determined in TEEs referred for ischemic stroke or transient ischemic attack (“stroke”) and compared with LASP prevalence in patients undergoing TEEs for other reasons (“non-stroke”). Stroke subtyping was performed using the Trial of Org 10172 in Acute Stroke Treatment (TOAST) criteria. There were 306 TEEs from 144 non-stroke and 162 stroke patients. Mean age and sex distribution were 56 ± 1 (mean ± SE) and 65% male in the non-stroke group and 58 ± 1 and 54% male in the stroke group. The overall prevalence of LASP was 31%. The prevalence of LASP was 28% (41/144) in non-stroke patients, 25% (24/95) in non-cryptogenic stroke patients, and 43% (29/67) in cryptogenic stroke patients. LASP prevalence was significantly higher in the cryptogenic subgroup compared with the non-cryptogenic subgroup (*p* = 0.02). These findings demonstrate a significant association of LASP with risk of cryptogenic stroke, suggesting that LASP may serve as a thromboembolic nidus. Additional studies are needed to determine the generalizability of these findings, and their therapeutic implications, supporting LASP as a stroke risk factor.

## Introduction

Cryptogenic strokes account for up to 30–40% of all ischemic strokes, and a significant subset is thought to originate from distant emboli despite exclusion of arrhythmias and intracardiac thrombus [1]. These infarcts are categorized as embolic strokes of undetermined source (ESUS), and their pathophysiology remains incompletely understood. Recent large, randomized trials comparing the efficacy of anticoagulation with antiplatelet therapy failed to demonstrate superiority of anticoagulation over aspirin in preventing recurrent stroke in patients with ESUS [2–4]. In these studies, presence or absence of structural or functional abnormalities of the left atrium that may promote thromboembolism independent of atrial arrhythmias was not examined. Indeed, left atrial dysfunction has been found to be more prevalent in ESUS than in other stroke subtypes [5, 6].

**Fig. 1:**
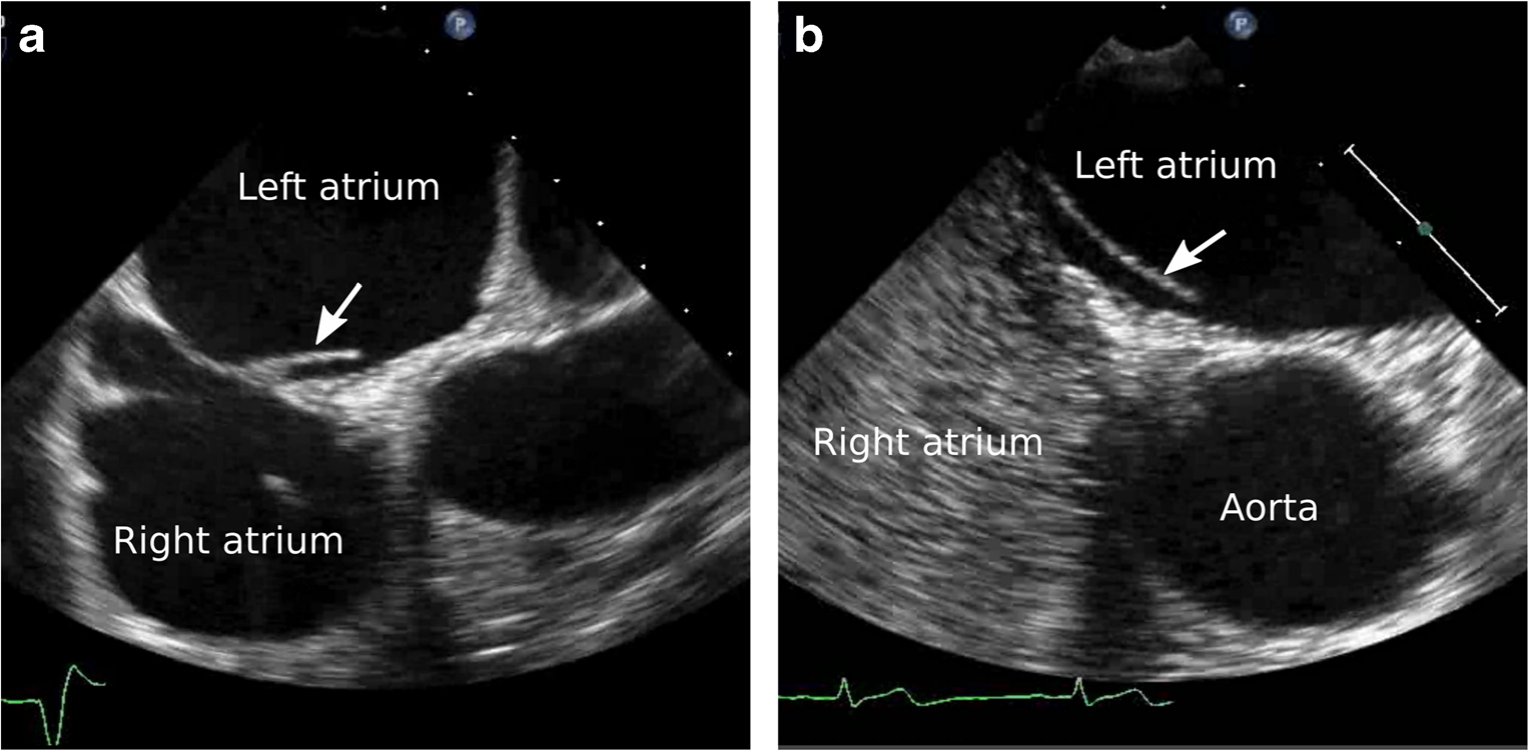
Examples of LASP (arrows) in our patient population. (**a**) A patient with an open flap directed towards the left atrium. (**b**) Absence of microbubbles in the left atrium demonstrates a fused septum in this patient with LASP

Examination of the left atrial structure may help elucidate a mechanism underlying ESUS or cryptogenic stroke. The anatomic variant known as the left atrial septal pouch (LASP) is of special interest as a possible source of emboli in cryptogenic strokes. LASP was first described in 2010 by Krishnan and Salazar as a unique anatomic entity that occurs with incomplete fusion of the inter-atrial septum [7]. In utero, a separation between septum primum and septum secundum allows for flow of oxygenated maternal blood into the fetal systemic circulation while bypassing pulmonary circulation. After birth, the left atrial pressure increases causing the gap between the septum primum and secundum to close and fuse over time. However, in 20–30% of adults, the two septa fail to fuse, leading to patent foramen ovale (PFO). PFO is particularly prevalent in the younger population and suggests that septal remodeling and fusion is a lifelong process [8]. As proposed by Hołda et al., a septal pouch results from partial fusion of a long PFO channel. This forms a diverticulum with a free edge or flap of one septum opening either into the right, left, or both atria [8]. LASP specifically results from fusion just at the caudal edge of the PFO channel, resulting in an opening towards the left atrium (Fig. 1). Nearly 40% of adults were observed to have LASP in the original study described by Krishnan and Salazar, although subsequent studies report varying prevalence of 11-42% [8]. The pouch-like structure of LASP can serve as a potential site of thrombus formation, especially in conditions prone to stasis in the left atrium, such as in atrial cardiopathy, atrial fibrillation, or mitral stenosis.

Since the initial reporting of LASP, a series of case studies have shown thrombus attachment to the LASP, including some instances in patients with strokes [9–11]. However, clearly linking the LASP with cryptogenic stroke has been more challenging. Results from previous retrospective studies have been mixed, with some showing a positive association between LASP and cryptogenic stroke [12–14] and others failing to demonstrate such a relationship [15–17]. Although a recent meta-analysis supports a positive association between LASP and cryptogenic stroke [18], interpreting the data collectively from these studies is limited due to multiple factors, including small sample sizes, heterogeneous patient populations, and widely varying LASP prevalence, which may reflect technical limitations of different imaging modalities used for visualizing the inter-atrial septum. The aim of the current study was to investigate the relationship between presence of LASP and stroke in a large, well-defined population of patients with stroke and control subjects who underwent transesophageal echocardiography (TEE).

## Methods

This was a cross-sectional retrospective study in which all patients who underwent consecutive TEE studies at the University of California Irvine Medical Center between July 2011 to December 2018 were screened for inclusion criteria. All consecutive TEE studies with the indication “stroke,” “CVA” (cerebrovascular accident), or “TIA” (transient ischemic attack) in the order requisition were included for this study in the “stroke group.” The “non-stroke group” was a subset of TEE studies from the same time period that did not specify “stroke,” “CVA,” or “TIA” as an indication. Examples of alternative indications included endocarditis, atrial fibrillation or flutter, or valvular disease. Incomplete TEE studies or TEE studies that did not include imaging of the inter-atrial septum were excluded. Patients with intracranial hemorrhage or control subjects with incidental finding of stroke on brain imaging were excluded from analysis. Patient charts were also reviewed for the presence of known vascular risk factors and comorbidities, including remote history of stroke or TIA, atrial fibrillation/flutter, congestive heart failure, coronary artery disease, diabetes mellitus, hypertension, hyperlipidemia, and smoking status.

The TEE images were evaluated for the presence of LASP, atrial septal defects (ASD), or PFO by a cardiology team composed of cardiology fellows and a senior medical resident, who were blinded to the patient demographics and other clinical information including stroke subtyping (inter-observer agreement 77–97%). An attending cardiologist and Director of the Echocardiography Laboratory (JKK), also blinded to patient information, confirmed the presence of LASP, and only in those with concordance with the original reviewer were included in the study. The presence of LASP was evaluated primarily in the bi-caval view (typically obtained with transducer in the mid-esophageal position and angled 90–110 degrees) or in the short-axis view (typically obtained with transducer in the mid-esophageal position and angled 25–45 degrees). LASP was defined as the fusion of septum primum and septum secundum at the caudal end with an open “flap” that created a septal pouch with the opening directed towards the left atrium, and the absence of ASD and PFO. ASD or PFO was excluded by evaluating the morphology of the inter-atrial septum in 2D views, color Doppler echocardiography, and agitated saline injection with and without Valsalva maneuver to confirm absence of inter-atrial shunt; as in prior studies [12–16], patients with ASD or PFO were excluded from the analysis. The presence of ischemic stroke was verified by review of head and neck imaging (magnetic resonance imaging, magnetic resonance angiography, and/or computed tomography angiography). All ischemic strokes were further subtyped by a vascular neurologist and by a cerebrovascular fellow in accordance with the modified Trial of Org 10172 in Acute Stroke Treatment (TOAST) criteria (inter-observer agreement 96%, kappa coefficient 0.92) [12, 19].

We compared the prevalence of LASP among non-stroke patients, patients with cryptogenic stroke, and patients with non-cryptogenic stroke. We compared differences in patient characteristics in non-stroke vs. stroke patients, LASP vs. non-LASP patients, and cryptogenic vs. non-cryptogenic stroke patients, using the chi-square test of proportions for categorical variables (Fisher’s exact test for 2 × 2 tables) and *t* tests for continuous variables. All tests were two-sided, and a p value < 0.05 was considered statistically significant. Univariate and multivariate odds ratio (OR) for stroke and cryptogenic stroke was determined in patients with LASP vs. no LASP. Multivariate analysis was adjusted for age, sex, hypertension, diabetes, hyperlipidemia, atrial fibrillation, coronary artery disease, congestive heart failure, and cardiac thrombus. Analyses were performed using SAS© version 9.4 (SAS Institute Inc., Cary, NC).

## Results

A total of 306 TEEs from 144 non-stroke and 162 stroke patients were evaluated for this study (Table 1). The non-stroke and stroke groups did not differ significantly by age (55.9 ± 1.2 vs. 58.2 ± 1.2 years old, *p* = 0.18) or sex (male 65% vs. 54%, *p* = 0.08). Stroke patients were more likely to have a history of hypertension (75% vs. 58%, *p* = 0.002). Non-stroke patients were more likely to have a history of atrial fibrillation or flutter (33% vs. 15%, *p* = 0.0002), congestive heart failure (24% vs. 9%, *p* = 0.0003), and coronary artery disease (22% vs. 12%, *p* = 0.045). The overall prevalence of LASP in our patient population was 31% (94 of 306). LASP was present in 41 (28%) non-stroke patients compared with 53 (33%) stroke patients (*p* = 0.46). Table 2 shows the patient characteristics by the presence or absence of LASP. There was no statistically significant difference between patients with and without LASP in regard to age, sex, or medical history.

**Table 1:** Characteristics of patients included in this study by stroke status

Characteristic	Non-stroke (*n* = 144)	Stroke (*n* = 162)	*p* value
Age—years (mean **±** SE)	55.9 ± 1.2	58.2 ± 1.2	0.18
Male sex—no. (%)	93 (65%)	88 (54%)	0.08
Medical history—no. (%)			
Hypertension†	84 (58%)	121 (75%)	0.002*
Diabetes mellitus	60 (42%)	60 (37%)	0.41
Hyperlipidemia†	56 (39%)	77 (48%)	0.13
Atrial fibrillation/flutter	47 (33%)	24 (15%)	0.0002*
Coronary artery disease	31 (22%)	20 (12%)	0.045*
Congestive heart failure	35 (24%)	14 (9%)	0.0003*
Prior stroke		35 (22%)	
Prior TIA		8 (5%)	
Cardiac thrombus—no. (%)	5 (3%)	8 (5%)	0.58
LASP—no. (%)	41 (28%)	53 (33%)	0.46

^†^One patient with missing history

^*^Statistically significant difference based on *p* < 0.05

**Table 2:** Characteristics of patients included in this study by presence/absence of LASP

Characteristic	No LASP (*n* = 212)	LASP (*n* = 94)	*p* value
Age—years (mean **±** SE)	56.9 ± 1.0	57.6 ± 1.6	0.72
Male sex—no. (%)	122 (57%)	59 (63%)	0.45
Medical history—no. (%)			
Hypertension†	143 (67%)	62 (67%)	0.90
Diabetes mellitus	83 (39%)	37 (39%)	1.00
Hyperlipidemia†	92 (44%)	41 (44%)	1.00
Atrial fibrillation/flutter	54 (25%)	17 (18%)	0.19
Coronary artery disease	35 (17%)	16 (17%)	1.00
Congestive heart failure	33 (16%)	16 (17%)	0.74
Prior history of stroke	25 (12%)	10 (11%)	0.85
Prior history of TIA	7 (3%)	1 (1%)	0.44
Cardiac thrombus—no. (%)	8 (4%)	5 (5%)	0.55

^†^One patient with missing history

**Fig. 2:**
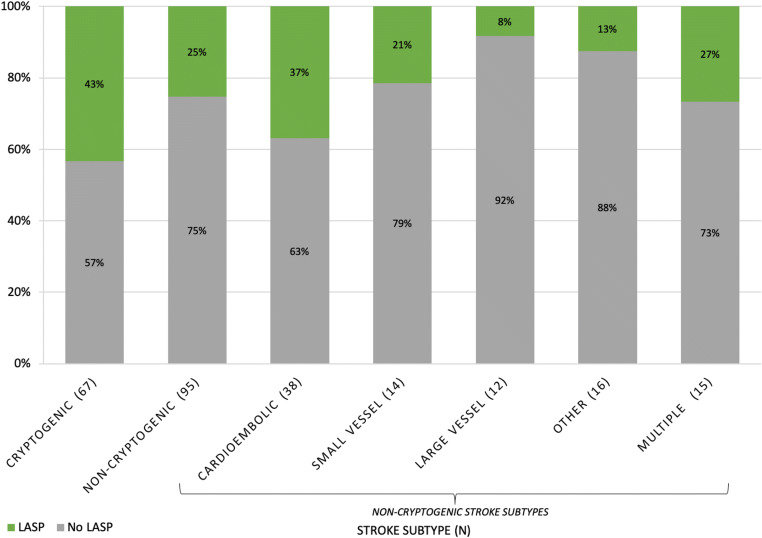
Prevalence of LASP in different stroke subtypes based on TOAST criteria

We further evaluated the relationship between LASP and different stroke subtypes based on the modified TOAST criteria (Table 3, Fig. 2). Of the 162 stroke patients in the study, 67 (41%) had cryptogenic stroke. Of the patients with cryptogenic stroke, 43% had LASP on their TEEs, which was significantly different from non-stroke patients (28%; *p* = 0.04). Cardioembolic stroke subgroup followed with the second-highest prevalence of LASP at 37%. LASP was least associated with large-artery atherosclerosis subtype. Among all non-cryptogenic stroke patients, the prevalence of LASP was 25%, which was significantly lower than 43% seen in the cryptogenic stroke subgroup (*p* = 0.02).

**Table 3:** Prevalence of LASP by stroke subtype

Stroke subtype	No LASP	LASP
Cryptogenic	38 (57%)	29 (43%)
Cardioembolic	24 (63%)	14 (37%)
Small vessel	11 (79%)	3 (21%)
Large vessel	11 (92%)	1 (8%)
Other	14 (88%)	2 (13%)
Multiple mechanisms	11 (73%)	4 (27%)
Cryptogenic	38 (57%)	29 (43%)*
Non-cryptogenic	71 (75%)	24 (25%)*

*p = 0.02 for difference in LASP prevalence between cryptogenic and non-cryptogenic stroke subgroups

Using univariate analysis, we found that the risk of stroke was increased in patients with LASP compared with patients without LASP, but not significantly so (OR 1.21; 95% CI 0.74–1.98). The results were similar when adjusted for common risk factors—age, sex, hypertension, diabetes, hyperlipidemia, atrial fibrillation, coronary artery disease, congestive heart failure, and cardiac thrombus (1.26; 95% CI 0.73–2.17). Using similar analysis, we found that the risk for cryptogenic stroke was significantly increased in the LASP population (OR 2.15; 95% CI 1.1–4.21) even after adjusting for common stroke risk factors (OR 2.37; 95% CI 1.13–4.98).

## Discussion

The current study is a retrospective evaluation of LASP in stroke and non-stroke patients who underwent TEEs and demonstrates a significantly increased risk of cryptogenic stroke in patients with LASP, even after adjusting for common stroke risk factors. The current study confirms and strengthens a prior investigation by our group, which demonstrated that cryptogenic stroke patients are more likely to have LASP compared with non-cryptogenic stroke patients [12]. Our two studies together support this anatomic entity’s pathological role in engendering thromboembolic strokes. Our findings are in disagreement with prior investigations that failed to show a correlation between LASP and cryptogenic strokes [15–17, 20]. However, some of those studies were significantly underpowered compared with the current study, used computerized tomography (CT) scan rather than TEE to evaluate the inter-atrial septum, or exclusively included older patients (> 50 years old) who are less likely to have LASP due to ongoing inter-atrial septal remodeling and fusion [8]. The largest study to date evaluated TEEs in 126 cryptogenic stroke and 184 non-stroke patients and demonstrated an overall LASP prevalence of 48% in their study population [14]. Similar to our results, they also show that patients with cryptogenic strokes were more likely to have LASP than non-stroke patients, but they did not compare cryptogenic stroke to other non-cryptogenic stroke subtypes.

It has been suggested that the structure of the LASP is in essence a smaller left atrial appendage which may predispose towards blood stasis and thrombus formation [9, 21]. Gurudevan et al. proposed that right upper pulmonary vein flow is laminar and thus generally protective against thrombus formation in the LASP until another predisposing factor (such as high filling pressures) disrupts the brisk pulmonary vein flow and leads to stasis near the inter-atrial septum. Most case reports demonstrating LASP thrombus are found in patients with another cardiac abnormality, such as valvular disease or left ventricular dysfunction, and thus are in agreement with the above hypothesis [9, 22–26]. In contrast, there are also isolated cases of stroke patients with LASP thrombus who lack predisposing factors [10, 11]. This suggests that the presence of LASP by itself may be a predisposing factor in thrombus formation and may suffice as a source of embolism in a subset of cryptogenic stroke patients. Interestingly, Hołda et al. note increased association of LASP with atrial fibrillation [27], whereas in our patient population, atrial fibrillation was more prevalent in patients without LASP (25% in patients without LASP vs. 18% in patients with LASP, see Table 2). The reasons for the different results are unclear as the overall prevalence of LASP (31% in our study vs. 36% in Hołda et al.) and atrial fibrillation (23% in our study vs. 36% in Hołda et al.) is similar in both studies. This warrants further investigation into whether LASP contributes to increased stroke risk by engendering atrial cardiomyopathy or arrhythmias.

The overall prevalence of LASP in this study (31%) is lower than the prevalence in autopsy studies (39% [7]; 47% [8]) but within the range determined by prior TEE-based studies (11% to 48% [18]) and comparable with studies using CT scan (29% [20]; 37% [28]). TEE-based evaluation of the LASP has the largest variation in prevalence estimates, likely related to operator and institutional differences in protocols used for image acquisition. Holda et al. evaluated the inter-atrial septum of 146 patients using TEE with different imaging planes and found good agreement between the bi-caval and short-axis views (kappa 0.68) [29]. They found that the bi-caval view was slightly more sensitive in detecting LASP than the short-axis view, though the difference was statistically insignificant. Elsayed et al. further demonstrated that 3D TEE, providing volumetric data otherwise unobtainable, was more sensitive in detecting LASP than 2D TEE [11]. These technical considerations are particularly important because most TEE-based evaluations of LASP, including this study, are retrospective where the primary intent of the echocardiographer at the time of the imaging may not have been to examine the inter-atrial septum for LASP per se. Not every TEE included both bi-caval and short-axis views, or a 3D interrogation of the inter-atrial septum, and it is likely that LASP may have been missed in some instances. While cardiac CT may have high resolution and utility in defining anatomy, an inter-atrial shunt cannot be excluded solely based on the CT-derived anatomic information. Thus, further research is needed to establish the most optimal noninvasive approach to evaluate LASP.

This study is subject to several limitations. Even though this is one of the larger studies to date that evaluates LASP in stroke patients, the number of patients in each subgroup is small and therefore limits our ability to draw conclusions about LASP prevalence in specific subtypes of non-cryptogenic strokes. Moreover, because this is a retrospective cross-sectional study, we are unable to investigate changes in LASP prevalence over time (i.e., ongoing remodeling of the inter-atrial septum as a person ages) or its direct impact on stroke risk.

In conclusion, the current study confirms results from our group and other studies. In combination with a recent meta-analysis, the data strongly suggest that LASP may be an independent risk factor for cryptogenic strokes [18]. Prospective, longitudinal studies are necessary to better understand the causality of LASP in cryptogenic stroke, further define the mechanism of LASP-related strokes, and investigate targeted treatment options for secondary stroke prevention.

## Data Availability

By request
